# App-technology to increase physical activity among patients with diabetes type 2 - the DiaCert-study, a randomized controlled trial

**DOI:** 10.1186/s12889-018-5026-4

**Published:** 2018-01-10

**Authors:** Stephanie E. Bonn, Christina Alexandrou, Kristin Hjörleifsdottir Steiner, Klara Wiklander, Claes-Göran Östenson, Marie Löf, Ylva Trolle Lagerros

**Affiliations:** 1 Clinical Epidemiology Unit, Department of Medicine Solna, Karolinska Institutet, Eugeniahemmet T2, 171 76 Stockholm, Sweden; 2 Department of Biosciences and Nutrition, Karolinska Institutet, Stockholm, Sweden; 3 Department of Neurobiology, Care Sciences and Society, Division of Family Medicine, Karolinska Institutet, Stockholm, Sweden; 4 Department of Molecular Medicine and Surgery, Endocrine and Diabetes Unit, Karolinska Institutet, Karolinska University Hospital, Stockholm, Sweden; 5 Clinic of Endocrinology, Metabolism and Diabetes, Department of Medicine, Karolinska Hospital Huddinge, Stockholm, Sweden

**Keywords:** Adults, Body composition, Exercise, HbA1c, Metabolic health, Randomized controlled trial, Smartphones

## Abstract

**Background:**

Physical activity can decrease the risk of complications related to diabetes type 2. Feasible and scalable strategies to implement support for a healthy lifestyle for patients in primary care are needed. The aim of the DiaCert-study is to evaluate a digital healthcare platform and the effect of a 12-week long smartphone-app physical activity intervention aiming at increasing physical activity (primary outcome) and improve levels of HbA1c (glycated hemoglobin), blood lipids, blood pressure, body composition, as well as other lifestyle factors and overall health in patients with diabetes type 2.

**Methods/Design:**

The DiaCert-study is a two-arm, randomized controlled trial that will include 250 patients with diabetes type 2. At baseline, participants are randomized 1:1 to intervention, i.e. use of the smartphone-app, during 12 weeks, or to a control group receiving only standard care. Physical activity and sedentary behavior, is objectively measured using the Actigraph GT3X. Biomarkers including HbA1c and blood lipids are measured in fasting blood samples. Anthropometrics include height, weight, waist circumference and body composition, and a number of lifestyle factors including sleep, diet, self-efficacy, and quality of life, are assessed through an extensive questionnaire. Measurements are made at baseline and at follow-up after 3, 6 and 12 months.

**Discussion:**

Using new technology, is one way to bridge the gap between what patients need and what health care can offer. This study evaluates a new digital health care platform and will show if use of a smartphone-app to promote daily steps is an effective and feasible method to increase physical activity and improve clinical markers in patients with diabetes type 2.

**Trial registration:**

ClinicalTrials.gov Identifier: NCT03053336; 7 Feb, 2017.

## Background

Lifestyle is important in the development of diabetes type 2 and a healthy such may prevent or delay a diagnosis [1, 2]. Nevertheless, globally, the total number of adults diagnosed with diabetes has quadrupled from approximately 100 million in 1980 to more than 400 million today [3]. Additionally, the proportion of adults with unhealthy lifestyles is increasing. In the Nordic countries, almost half of all adults were reported to be overweight or obese, and one third was classified as physically inactive, i.e. not meeting recommendations of moderate-to-vigorous physical activity as defined in the Nordic Nutrition Recommendations 2012 [4], in 2014 [5].

Lifestyle factors, such as overweight or obesity, having an unhealthy diet, or being physically inactive, may also increase the risk of complications from the diabetes. Complications can be serious and may include cardiovascular disease, damage to the kidneys and eyes, feet ulcers leading to amputations, and even premature death [3]. A healthy lifestyle can, on the other hand, decrease the risk of complications related to diabetes type 2. Higher levels of physical activity have been associated with a lower risk of cardiovascular disease, microvascular events, and premature death in patients with diabetes type 2 in observational studies [6, 7].

Early use of technology, such as use of computers, i.e. websites, or mobile phones with short text message services, has been shown to have positive effects on the level of physical activity in patients with diabetes type 2 [8, 9]. However, with the rapid technology development of today, smartphone-applications (apps) are now available for large scale use. Instead, smartphone-apps have been used successfully to improve lifestyle behaviors and clinical outcomes [10]. In Sweden, over 80% of the population own a smartphone and ownership, as well as usage, is independent of socioeconomic status [11]. Use of new technology could be a way to reach large numbers of patients independent on socioeconomic status.

In a review by Schoeppe et al. [10], studies using app-strategies targeting physical activity specifically showed improvements in activity, but although some studies included overweight and/or obese individuals, none included patients with diabetes type 2. Previous studies using app-strategies in patients with diabetes type 2 have mostly focused on glucose monitoring alone, or in combination with lifestyle advice on for example diet and physical activity [12]. Two smartphone-apps that included a physical activity component also included several other components such as, for example, measurement and monitoring of blood glucose, food habits [13], as well as additional monitoring of blood pressure and body weight [14].

Patients with diabetes type 2 are generally taken care of by the primary care and one of the most effective strategies to improve health is regular visits to health clinics [15]. However, this is not a feasible strategy given the large and growing number of patients. New technology, such as eHealth, can be used as an additional strategy to reach patients. eHealth is, as defined by the World Health Organization (WHO), the use of information and communication strategies for health. An additional component of eHealth is mHealth, which has been defined by the WHO as a medical or public health practice that is supported by mobile devices [16]. Mobile devices may include, for example, mobile phones, patient monitoring devices, personal digital assistants, and other wireless devices. Using mHealth strategies, i.e. utilizing the Internet and smartphones, could be one way to bridge the gap between what patients need and what health care can offer. To meet both the needs of patients and the capacity of the health care system, novel ways to implement lifestyle changes are needed.

### Aim

The aim of this paper is to describe the study design and methodology of the DiaCert-study. The main aim of the trial is to evaluate the effect of a 12-week long smartphone-app physical activity intervention aiming at increasing physical activity (primary outcome) and improving levels of HbA1c (glycated hemoglobin), blood lipids, blood pressure, body composition (secondary outcomes), as well as other lifestyle factors and overall health in patients with diabetes type 2.

### Hypothesis

The hypothesis is that the smartphone-app intervention to promote daily physical activity will be effective in increasing physical activity, improving cardiovascular risk factors and other lifestyle factors after 12 weeks of active intervention and long term at 6 and 12 months of follow-up in the intervention group compared to the control group.

## Methods

### Study design and recruitment

The DiaCert-study is a randomized controlled trial with two parallel arms. Patients fulfilling the inclusion criteria are recruited from five primary health care centers around Stockholm, Sweden. Each primary care center volunteered to participate in the study and data collection is performed in collaboration with clinicians and nurses at the centers. Patients that sign up for the study at the health care clinics are contacted by phone. During that contact, patients are given more information about the study. If they fulfill the inclusion criteria and agree to participate they are booked for a meeting in person at which they give their signed informed consent to participate, respond to a lifestyle questionnaire and have baseline measurements taken.

**Fig. 1:**
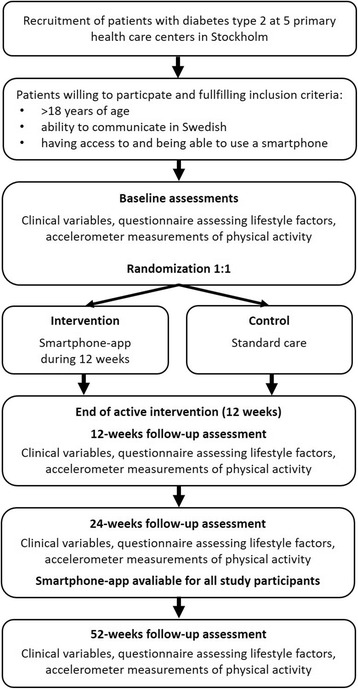
Flow-chart of the DiaCert-trial study design

At baseline, study participants are randomized 1:1 to the intervention or control group. All participants, both in the intervention and the control group will continue to receive standard care, i.e. continue their usual care as prescribed by their primary care giver at each participating center, during the whole study period. For participants in the intervention group, the active intervention, i.e. use of the smartphone-app, last for 12 weeks from baseline. All study participants will meet with study personnel at baseline and are followed-up after the intervention at 3 and 6 months, and again after 1 year for assessment of clinical and life style factors. Physical activity is measured using the Actigraph GT3X at baseline and follow-up. For ethical reasons, participants in the control group are offered to use the smartphone-app at the 6-month follow-up if they want. Participants in the intervention group will be offered to download the app again at the same occasion if they wish to start using it again. The study design is outlined in Fig. 1.

The intervention is described according to the CONSORT statement [17] and the CONSORT-EHEALTH checklist developed specifically for eHealth/mHealth interventions [18].

### Inclusion and exclusion criteria

Both women and men are eligible for participation. Inclusion criteria are: having a diagnosis of diabetes type 2, being above the age of 18 years, ability to communicate in Swedish, and having and being able to use a smartphone. Exclusion criteria are: not being able to walk.

### Randomization and blinding

Study participants are randomized to either intervention group (standard care and use of the DiaCert smartphone-app) or control group (only standard care) at baseline at a 1:1 ratio by means of a random allocation sequence list generated in STATA 14.0. Women and men are randomized separately in blocks of ten within each participating primary health care center. Participants are, however, not blind to their allocation due to the nature of the intervention.

### Intervention

#### Overall

The aim of the intervention is to achieve higher levels of physical activity and subsequent improvements in clinical variables including HbA1c, fatty acids, anthropometric variables, and lifestyle factors, through use of a digital platform (for the care giver) and smartphone-app (for the patients) during 12 weeks. The app is compatible with both Android (version 4.1 or higher) and iOS (version 9.2 and higher).

#### Download

The intervention group will download the DiaCert smartphone-app which is connected to a digital platform where the care giver can follow the activity (daily steps) of each individual user. The app is demonstrated and downloaded at the introductory baseline meeting. An individual user account for each study participant is created by the study personnel on the digital platform where data is stored. Unique and temporary 6-digit codes are used to ensure a secure connection to the app for each participant. The individual access codes are created by study personnel and communicated to participants via telephone 7 days after the introductory meeting to avoid overlap with baseline accelerometer measurements. Daily steps measured by the smartphone are thereafter registered and displayed automatically in the app.

After 12 weeks of usage, participants in the intervention group are asked to remove the app from their phones and respond to a short questionnaire including 16 questions about using the app. At the 6-month follow-up, all participants (both in the intervention and control group) are offered to download and connect to the app.

### The DiaCert smartphone-app

#### Individual step-goal

When study participants are connected to the DiaCert-app, an individual step-goal between 1000 and 10,000 steps is set together with study personnel. The step goal is set in discussion with each participant based on their usual activity level. The goal is thereafter revised every two weeks. Participants are contacted by study personnel and given the opportunity to increase their step goal by an even 500 steps if they wish, e.g. participants may increase their goal by, 500, 1000, 1500, 2000, etc. steps at revision. They may also keep the same goal. The maximum goal set at baseline or during follow-up is 10,000 steps.

#### Feedback and presentation of steps

Each day where the goal is achieved, the user will receive a positive feedback message saying “Well done, you have reached your step-goal”. Days when the goal is fulfilled are also marked in the app. On the first page of the app, steps from the last 7 days are visually displayed in circles that “fill-up” during the day as the user works towards the total daily step goal. When the goal is reached the circle has changed from empty (white) to filled (completely blue) and is marked with a check mark. The average number of total daily steps during the last 7 days is also shown here. Further, users can follow their daily steps from all days during which the app has been used in a bar chart. A day when the step goal is fulfilled is displayed as green bar in the chart while days when the goal is not fulfilled are displayed as red bars in the same chart.

#### Display of HbA1c levels

In the DiaCert-app, patients can also follow their HbA1c levels. Data is entered manually into the app for each participant by study personnel. Information on HbA1c analyzed within the intervention are displayed (date and HbA1c mmol/mol) in a chart in the DiaCert-app.

### Sample size and power considerations

Power calculations were performed prior to study start to ensure sufficient power to detect a clinically significant difference in the primary outcome (accelerometer measured physical activity). A total of 200 patients (100 in each group) completing the study would provide sufficient statistical power (80%) at the two-sided 5% significance level to detect a difference in moderate-to-vigorous activity of 8 min/day (e.g. 22 min/day vs. 30 min/day). A standard deviation of ±20 min/day of moderate-to-vigorous activity was assumed in both groups. To cover for a 20% dropout rate (based on earlier intervention studies conducted by our research group), we will recruit 250 patients (125 in each group) to the trial.

### Outcome measures

Objectively measured physical activity using accelerometers is the primary outcome for the intervention. Secondary outcomes include levels of HbA1c, plasma lipids, blood pressure, body composition, as well as other lifestyle factors and overall health in patients with diabetes type 2.

#### Physical activity

Physical activity and sedentary behavior is assessed using the Actigraph wGT3x-BT triaxial accelerometer (Actigraph Corporation, http://actigraphcorp.com/). To increase feasibility and maximize compliance with accelerometer measurements [19], participants are asked to wear the accelerometer on their non-dominant wrist during all hours for seven consecutive days at baseline, and at 3, 6, and 12-months follow-up. Data is sampled at a frequency of 80 Hz. Participants are also asked to keep a log over their non-wear time, e.g. when performing water activities or removing the accelerometer for other reasons. The accelerometer and log-book is mailed back to study personnel in pre-paid envelopes.

Additionally, data on daily steps will be collected from the DiaCert-app.

#### Biomarkers

HbA1c (mmol/mol), triglycerides (mmol/L), total cholesterol (mmol/L), low-density lipoprotein (LDL)-cholesterol (mmol/L) and high-density lipoprotein (HDL)-cholesterol (mmol/L) are measured in fasting blood samples at baseline and follow-up, at 3, 6, and 12 months. HbA1c is measured using the IFCC (International Federation for Clinical Chemistry and Laboratory Medicine) reference measurement procedure [20, 21]. Total cholesterol, triglycerides and HDL-cholesterol are measured using the enzymatic method. LDL-cholesterol is calculated from levels of triglycerides, total cholesterol and HDL-cholesterol using Friedewalds equation; LDL-cholesterol (mmol/L) = total cholesterol – HDL-cholesterol – (0.45*triglycerides). The ratio LDL/HDL-cholesterol is calculated from LDL- and HDL-cholesterol levels.

#### Body composition and blood pressure

Body weight (kg) and waist circumference (cm) are measured by study personnel at baseline, and follow-up at 3, 6, and 12 months. Weight is measured to the nearest 0.1 kg in light clothes with no shoes. Waist circumference is measured around the waist 2 cm above the umbilicus to the nearest cm using a non-stretchable SECA 201 tape. Height is measured at baseline to the nearest 0.5 cm. Body composition, including percentage body fat and fat free mass, is assessed at baseline and 3, 6, and 12-months follow-up at two of the participating primary health care centers. Body composition is measured using a Tanita digital body composition analyzer (Model BC-418), utilizing an 8-electrode bioelectrical impedance analysis with current going from foot-to-hand and hand-to-foot on both sides of the body.

#### Blood pressure

Blood pressure (systolic and diastolic) is measured manually by study personnel and using two electronic monitors with Bluetooth technology (Beurer BM85 and Andersson BDR 2.0). Measurements are done with participants in a seated position with legs uncrossed after the participant has been seated for at least 10 min.

#### Questionnaires

Study participants respond to paper-based questionnaires at baseline, and follow-up at 3, 6, and 12-months. The questionnaire takes approximately 60 min to complete.

*Participant characteristics* are collected using questions on civil status, education level, income, current occupation, tobacco use (smoking habits and snuff use), and diabetes related medication use. Weight and height are also self-reported prior to measurements at baseline. Smartphone use (hours/day) is assessed and participants are asked to estimate how often they bring their phone with them when they go out walking.

*Sleeping habits* are assessed using a 13-item long version of the Karolinska Sleep Questionnaire [22, 23] assessing sleeping time the previous night, stress and anxiety at time of going to bed, number of awakenings during the night and sleep quality.

*Subjectively reported physical activity* is assessed with two questions used in routine care of patients with diabetes type 2 [24]. Participants are also asked to report their time spent sitting, performing household chores and if they have been exercising during the past seven days. Participants who report to having exercised are asked about the type of activity performed and frequency and duration of performing that activity. Specifically, weight lifting, aerobics, swimming or water aerobics, and cycling are assessed. Participants can also report “other” activities performed. Additionally, participants’ intentions to change their physical activity habits are also assessed.

*Social support for being physically active* is assessed using a 5-item questionnaire adapted from the Physical Activity Social Support [25]. General support, as well as support from friends, family and colleagues is evaluated.

*Stress* is measured using the Perceived Stress Scale originally developed by Cohen et al. [26]. In the 14-item questionnaire, participants are asked to respond to how often (ranging from “never” to “very often” on a 5-point Likert scale) they perceive to react in a number of stressful situations.

*Neighborhood environment* is assessed using an adapted version of the Neighborhood Environment scale [27]. In total, 17 items including accessibility and facilitators for being physically active, such as safety, traffic and social environment in the neighborhood are included.

*Dietary intake* is measured using a validated semi-quantitative food frequency questionnaire (FFQ) [28]. Participants are asked to report, how often, on average, they consume each food and beverage, including alcohol, in the FFQ. Five additional questions used in clinical practice, developed by the Swedish National Food Agency assessing overall dietary habits are also included [29]. Drinking habits are further assessed in two questions assessing amount and intensity of consumption of alcohol [24].

*Diabetes self-efficacy and distress* is assessed using the Swedish translation of the Problem Area in Diabetes Questionnaire (Swe-PAID-20) [30]. In this 20-item questionnaire, participants rate their distress with having diabetes on a five-point Likert scale ranging from “Not a problem” to “Serious problem”.

*Health Related Quality of Life* is assessed using RAND-36 [31]. RAND-36 includes 36 questions within eight different domains and responses can be classified into a physical component summary scale and a mental component summary scale. Lastly, *purpose in Life* is assessed using a Swedish translation of The Life Engagement Test including six questions [32].

### Statistical analysis

Descriptive statistics will be used to describe characteristics of the study population at baseline. If differences in baseline characteristics are detected, potential confounders will be taken into consideration in further analysis. Data will also be checked for missing values, outliers and normality.

We will summarize pre-specified outcome measures assessed at 12 and 24 weeks of follow-up and compare intervention and control group assessing differences between groups using t-tests, ANOVAs and logistic regression. Analysis will be made following the intention-to-treat approach. Moreover, after completion of the study, follow-up assessment at week 52 will be analyzed within and between intervention and control groups to assess long-term effects. To assess the effect of both time and intervention on the outcomes, trends over time in outcomes will be analyzed using generalized estimation equations. Statistical interaction effects between characteristics and the intervention will also be tested for. Models will be adjusted for unbalanced baseline variables detected. We will also perform analysis stratified on age, sex, BMI, waist circumference, country of birth and primary health care center.

Additionally, results of usage and participants’ opinions regarding usability and satisfaction with the smartphone-app assessed using 16 questions asked at the 3-months follow-up, after the end of the active intervention, will be summarized in the intervention group and investigated in association to baseline factors, using linear and logistic regression.

### Trial status

The first study participants were recruited into the trial in February 2017. Data collection is ongoing with recruitment and baseline assessment planned to continue until a total of 250 study participants have been included or by the end of June 2018, whichever comes first. Follow-up assessments will be complete one year after the last study participant has been recruited.

## Discussion

The DiaCert-study aims to evaluate the effect of a smartphone-app intervention primarily targeting physical activity in patients with diabetes type 2. The randomized controlled design and large sample size are notable strengths to the study. Study participants are being recruited among patients at five different primary care centers located in areas with different populations and levels of socioeconomic status, increasing the generalizability of our results to different patient groups. Nevertheless, the inclusion criteria of being able to communicate in Swedish is a limitation to the study, as patients with limited knowledge of Swedish are more prevalent in areas of lower socioeconomic status. Another limitation is the inclusion criteria of having a smartphone. However, smartphone usage in Sweden is high (>80% of adults) and independent of socioeconomic status [11]. A strength is that the smartphone app is developed for usage on both Android and iOS devices which increases feasibility of the app as most smartphone users can download and use it. Careful calculations of statistical power a priori and objective assessment of outcomes, e.g. accelerometer measured physical activity, measured anthropometric and clinical variables are also notable strengths of this study.

Increased physical activity has been associated with a decreased risk of complications from the disease and premature death [6, 7]. Interventions targeting physical activity, but not using app-technology, have been successful in increasing physical activity levels among patients in primary care in general [33]. A pedometer-based program including telephone, but not smartphone, support has also showed positive effects on physical activity levels in patients with diabetes type 2 [34]. App-strategies targeting physical activity have been shown successful in increasing activity levels in previous studies [10], however, no apps specifically targeting physical activity have been developed for and evaluated in patients with diabetes type 2. DiaCert has been developed with both patients and the health care system in mind. Patients share personal data on physical activity (steps) with their caregiver and receive information on HbA1c directly from the health care system in their smartphone. It is a novel solution of data sharing including a smartphone-app for the patients and a digital platform for the health care personnel.

## Abbreviations

App: Smartphone-application
FFQ: Food frequency questionnaire
HbA1c: Glycated hemoglobin
HDL: High-density lipoprotein
IFCC: International Federation for Clinical Chemistry and Laboratory Medicine
LDL: Low-density lipoprotein
Swe-PAID-20: Swedish translation of the Problem Area in Diabetes Questionnaire
WHO: World Health Organization
